# Factors and predictors of length of stay in offenders diagnosed with schizophrenia - a machine-learning-based approach

**DOI:** 10.1186/s12888-020-02612-1

**Published:** 2020-05-06

**Authors:** Johannes Kirchebner, Moritz Philipp Günther, Martina Sonnweber, Alice King, Steffen Lau

**Affiliations:** 1 University Hospital of Psychiatry Zurich, Department of Forensic Psychiatry, Zurich, Switzerland; 2 University Hospital of Psychiatry Zurich, Department of Psychiatry, Psychotherapy and Psychosomatics, Zurich, Switzerland

**Keywords:** Forensic psychiatry, Schizophrenic offenders, Length of stay, Machine learning, Patient characteristics

## Abstract

**Background:**

Prolonged forensic psychiatric hospitalizations have raised ethical, economic, and clinical concerns. Due to the confounded nature of factors affecting length of stay of psychiatric offender patients, prior research has called for the application of a new statistical methodology better accommodating this data structure. The present study attempts to investigate factors contributing to long-term hospitalization of schizophrenic offenders referred to a Swiss forensic institution, using machine learning algorithms that are better suited than conventional methods to detect nonlinear dependencies between variables.

**Methods:**

In this retrospective file and registry study, multidisciplinary notes of 143 schizophrenic offenders were reviewed using a structured protocol on patients’ characteristics, criminal and medical history and course of treatment. Via a forward selection procedure, the most influential factors for length of stay were preselected. Machine learning algorithms then identified the most efficient model for predicting length-of-stay.

**Results:**

Two factors have been identified as being particularly influential for a prolonged forensic hospital stay, both of which are related to aspects of the index offense, namely (attempted) homicide and the extent of the victim’s injury. The results are discussed in light of previous research on this topic.

**Conclusions:**

In this study, length of stay was determined by legal considerations, but not by factors that can be influenced therapeutically. Results emphasize that forensic risk assessments should be based on different evaluation criteria and not merely on legal aspects.

## Background

In recent years, prolonged inpatient treatment in general and forensic psychiatry in particular have faced more and more criticism and scientific scrutiny: Especially within involuntary treatment settings, inappropriately long stays have been viewed as potentially unethical [1–6]. In addition, doubts have been raised about the benefits of prolonged inpatient treatment for patients’ rehabilitation [3, 7]. Prolonged duration of inpatient treatment has been discussed as an indicator of economic inefficiency - particularly for forensic inpatient treatment, which constitutes a low-volume high-cost sector [3, 8–14]. The internationally observed prolongation of forensic hospitalizations in the past years [1, 3, 7, 15–17], as well as the ever-growing demand for forensic services [18–21], have become a subject of socio-political debate with urgent need for more research on avenues to reduce the duration of inpatient treatments in order to reduce exploding costs whenever possible [2, 4]. A recent review of 38 studies in eleven countries summarized a rich set of patient characteristics contributing to length of stay in psychiatric inpatient treatment [6], but concluded that just ten studies were useful in identifying clinically useful predictive factors, since “more rigorous multivariate statistical techniques” are required in order to eliminate confounding factors. Its authors also conducted an extensive qualitative and quantitative exploratory inquiry of the topic drawing on information from all stakeholders (patients, treatment professionals, experts) and mentioned not conducting file reviews on long-stay versus non-long-stay patients in forensic psychiatry using adequate sophisticated statistical tools as a key limitation to their comprehensive work. The present study aims to fill this gap using machine learning – a statistical approach novel to the field of psychiatry, which has recently been identified as superior in direct comparison to contemporary statistical approaches such as binary regression analysis in its sensitivity, specificity, accuracy and predictive validity [22]. Machine learning (ML) is a sub form of artificial intelligence and relies on patterns and inference in a set of data in order to find an algorithm best predicting an outcome (such as length of stay in the present study). In exploratory data analysis it is therefore better suited than conventional statistical methods to uncover previously “invisible” non-linear dependencies between variables, often also resulting in better predictive power [23, 24].

By - to our knowledge - applying machine learning for the first time to the investigation of predictors of length of stay in forensic psychiatric institutions, the current study should help to better meet the statistical requirements of this complex and non-linearly related data set [6] and thus resolve inconsistencies of previous findings on this topic. These will be summarized in the remainder of this section along with frequently confirmed prior findings, since they have informed the primary set of variables explored in the present study. Furthermore, we provide a brief overview of the legal requirements for forensic psychiatric admissions and discharges in Switzerland, as these can vary greatly from one country to another and represent an important aspect that informs clinical release recommendations.

### Findings and inconsistencies of relevant prior research

Past researchers studied patients from different security settings [25–27] or regardless of their moving (or not moving) from one level of security to another [1, 15, 16, 28, 29]. In some research, factors which were found to be relevant to patients’ transfer from a medium to a minimum security setting were set equal to those relevant to patients’ discharge into the community, and vice versa [27]. Furthermore, studies usually did not limit their sample to patients of a specific legal status [3, 14, 30, 31]. Since different requirements for discharge apply due to different legal verdicts, it may well be that factors associated with duration of inpatient treatment also differ accordingly.

Studies revealed considerable differences in duration of forensic hospitalization between countries, and even between different regions within countries, suggesting substantial geographical variation in treatment standards, structural conditions of forensic care, as well as legal procedures [11, 16, 29, 32]. Switzerland, the setting of the present study, is not among the 11 countries in which length of stay has been explored so far [6], thus providing new information on geographical inconsistencies.

With regard to socio-demographic factors, factors correlating with prolonged inpatient treatment included male gender [3, 33, 34], white skin colour [25, 30, 34], advanced age at the time of admission [15, 28], being unmarried [34, 35], low educational qualifications [16, 28, 34–36], low IQ [35], adjustment, socialisation, and partnership issues [36], no discharge address [15], unemployment before admission [16, 28, 35–37], and having lived with ones parents before admission [16]. There is also some evidence that emotional neglect during childhood has a prolonging effect [7]. Socio-demographic variables associated with a reduction of time spent in inpatient treatment included being a parent [1], good contact with one’s family or good social support [26–28], and living in a close relationship [16]. While some studies reported prolonged inpatient treatment for certain religious minorities [28] and patients having migrated [7], others reported shorter length of stay for immigrants [16] and ethnic minorities [17].

Regarding patients’ criminal histories, empirical research indicated patients being forensically hospitalized for a prolonged period of time to be more likely to have engaged in past criminal and violent behaviors [3, 26, 35] and to be of younger age at their first delinquency or violent incident [3, 16, 35]. Patients who had been admitted to a (forensic) psychiatric institution before or had been younger at their first psychiatric contact also tended to be hospitalized longer [1, 7, 16, 17, 31, 34, 38]. By contradiction, other studies [15, 39] reported patients who had previously been admitted to a forensic psychiatric hospital to have shorter hospitalizations.

With respect to the index offence leading to forensic hospitalization, researchers recurrently reported the severity of the offence to be an important factor and predictor for inpatient treatment duration. The more serious the index offence, the longer the patient’s hospitalization [15, 16, 25, 28–31, 33–36, 38–41]. Additionally, studies suggested factors such as having committed a violent index offence [1, 17, 39], having been young at the time of the index offence [37], having offended against multiple victims [34], and having committed the offence against someone known to the patient [35] also extend forensic hospitalization.

In terms of clinical assessment tools, lower “Global Assessment of Functioning” scores [1, 42], lower “Positive and Negative Syndrome Scale” scores [28], psychotic symptoms [27, 43], psychotic vulnerability, being in need of psychiatric medication [7], and having no insight into the mental illness [27] correlated with prolonged forensic inpatient treatment. Other studies, limiting their studied sample to offender patients with a schizophrenia spectrum disorder, suggested the presence of positive symptoms may have a protective effect against long hospitalization times [15, 37]. A history of substance abuse [3, 7, 15, 44], a comorbid medical illness [28], and a learning disability [15] correlated with the duration of forensic hospitalizations.

In terms of forensic treatment variables, adverse behaviors and events such as violence, substance abuse, absconding, non-compliance, requirement of seclusion, physical restraints, forced medication, or conditional release failure significantly delayed discharge [1, 3, 16, 26, 27, 31, 33, 35, 38, 42]. Patients who stayed hospitalized for a shorter period of time were more likely to make good therapeutic progress [15, 26], participate in more therapy programmes [26], work in the hospital [28], reside in open wards, have higher levels of ground privileges, be involved in community, educational, or vocational activities [42], participate in activities in general [27], are more likely to be cooperative [29], express remorse for their crime(s), and have positive references [35]. All variables investigated in the present study are shown in Table 1 and are described more detailed in the Additional file 1.

**Table 1:** Variables explored in current study and prior research

variable in current study	categorization in current study	prior research with similar variable
*Socio-demographic variables*		
age at admission	numerical	[1, 6, 14, 16, 17, 26–30]
gender	dichotomous (female, male)	[1, 5, 6, 15, 16, 26, 27, 30, 34, 42]
marital status at time of index offence	dichotomous (married, single)	[5, 6, 15, 16, 26–28, 31, 35]
level of education	dichotomous (graduation from mandatory schooling/ no graduation from mandatory schooling)	[1, 6, 14, 16, 28, 34, 35]
employment at time of index offence	unemployed/ employed/ other (retired…)	[5, 6, 16, 26, 27, 35]
country of birth Switzerland	dichotomous (yes/ no)	[5–7, 14, 16, 17, 26–28, 30, 34]
religion	Catholic/Muslim/other	[6, 28]
homelessness at index offence	dichotomous (yes/ no)	[1, 15, 16]
*Childhood variables*		
childhood history of physical abuse	dichotomous (yes/ no)	[5]
childhood history of sexual abuse	dichotomous (yes/ no)	[7]
relationship instability in childhood	dichotomous (yes/ no)	[5, 7, 17]
parental psychiatric history	dichotomous (yes/ no)	[5, 7]
sexual deviation in childhood	dichotomous (yes/ no)	[7]
psychiatric admission in childhood	dichotomous (yes/ no)	[5, 6]
alcohol abuse in childhood	dichotomous (yes/ no)	[5, 35]
school maladjustment	dichotomous (yes/ no)	[5, 35]
poor family socioeconomic status (according to the definition of poverty by the Swiss Federal Statistical Office 2016 [45])	dichotomous (yes/ no)	[35]
childhood aggression	dichotomous (yes/ no)	[5, 35]
separation from caregiver	dichotomous (yes/ no)	[5, 35]
*Offence related variables*		
index offence leading to admission	homicide, including attempted/assault/threat, coercion/sexual abuse of children/rape, sexual assault/other sexual offence/ property crime without violence/property crime with violence/arson/criminal damage/traffic offences/offences against the controlled substances act/offences against the weapons act/other offences	[1, 5–7, 14–17, 27–31, 34, 35]
age at index offence	numeric	[1, 5–7, 35]
number of index offences	numeric	[6, 35]
any previous conviction	dichotomous (yes/ no)	[5–7, 14–17, 28, 30, 31]
age at first conviction	numeric	[5–7, 16, 17, 30]
victim injured deadly/severely	dichotomous (yes/ no)	[34, 35]
number of victims	numeric	[34]
victim known to offender	dichotomous (yes/ no)	[34, 35]
alcohol involved at offence	dichotomous (yes/ no)	[42]
previous forensic psychiatric admissions	dichotomous (yes/ no)	[15–17]
withdrawal of conditional release	dichotomous (yes/ no)	[16]
residual criminal responsibility	dichotomous (yes/ no)	[6, 16]
*Psychiatric variables*		
number of previous psychiatric admissions	numeric	[5, 6, 14–17, 30, 31, 35]
age at first admission	numeric	[14, 16, 30]
specific schizophrenic spectrum disorder	schizophrenia/schizotypal disorder/acute psychotic disorder/schizoaffective disorder	[1, 5–7, 14–17, 27, 28, 30, 31, 35, 42]
personality disorder lifetime	dichotomous (yes/ no)	[1, 5, 6, 14–17, 27, 30, 31, 35]
mood disorder lifetime	dichotomous (yes/ no)	[1, 5, 6, 14–16, 27, 28, 42]
alcohol abuse lifetime	dichotomous (yes/ no)	[6, 15, 17, 31, 35]
substance abuse lifetime	dichotomous (yes/ no)	[5–7, 15–17, 27, 28, 31]
sexual deviation	dichotomous (yes/ no)	[5, 7]
level of intelligence	high/average/low	[6, 7, 16, 35]
history of self-harming	dichotomous (yes/ no)	[6]
history of suicide attempt(s)	dichotomous (yes/ no)	[6]
delusions prior to admission	dichotomous (yes/ no)	[5, 27]
hallucinations prior to admission	dichotomous (yes/ no)	[5, 27]
cognitive impairment prior to admission	dichotomous (yes/ no)	[6]
*Current forensic hospitalization*		
violent incidents	dichotomous (yes/ no)	[1, 5, 6, 16, 31, 42]
self-harming	dichotomous (yes/ no)	[6]
suicide attempt(s)	dichotomous (yes/ no)	[6, 16, 42]
attempts to substance use	dichotomous (yes/ no)	[1]
escape attempt(s)	dichotomous (yes/ no)	[1, 6, 16]
performance in occupational work	good/average/low	[6, 26]
difficulties during psychological therapy	dichotomous (yes/ no)	[6]
pharmacological treatment	dichotomous (yes/ no)	[6, 7, 15]
contact with family/friends	dichotomous (yes/ no)	[6]
changes in diagnosis during current treatment	Dichotomous (yes/ no)	[6]
admission source into current hospitalization	none/supervised living facility/prison/other forensic hospital/psychiatry	[6, 30]
involuntary medication	dichotomous (yes/ no)	[31]
insight into wrongfulness of offence	dichotomous (yes/ no)	[35]
adherence (insight into illness and need of therapy)	dichotomous (yes/ no)	[26]
PANSS at admission	numeric	[28, 29]
PANSS at discharge	numeric	[28, 29]

### Legal requirements for admission and release from forensic psychiatric treatment in Switzerland

Patients enrolled in this study were admitted for “treatment of a mental disorder” in a forensic psychiatric facility according to Article 59 of the Swiss Penal Code, which means that they had committed a crime that is related to a mental disorder and that an expert opinion has concluded that psychiatric treatment can reduce the risk of future crimes. The necessity for this forensic psychiatric measure is reviewed annually by the referring authority. If it is ascertained that the offender’s risk of future offences has been sufficiently reduced, the offender is released from the measure. If the treatment lasts longer than 5 years, the decision of the authority is additionally reviewed by a court and may base its decision on a new external assessment. A release from inpatient treatment is granted if the hospital’s practitioners state that the treatment was successful and the referring authority shares this assessment. The assessment of the hospital’s practitioners is based on a clinical evaluation process, which also incorporates the results of established prognosis instruments**.**

### Objectives

The objectives of this exploratory study were to analyse the length of stay using machine learning (1) based on the unique group of forensic offender patients with schizophrenia spectrum disorder, (2) to consider all variables used in previous research on the subject, (3) to identify the most influential of these variables, and (4) to quantify a predictive value to distinguish between long and short stay.

## Methods

### Setting

This empirical study was conducted in a Swiss forensic psychiatric hospital, the Center for Inpatient Forensic Therapy which is part of the Clinic for Forensic Psychiatry at the Psychiatric University Hospital of Zurich. With a total of 79 available beds, the institution is committed to providing inpatient treatment for judicially admitted mentally disordered offenders, as well as for imprisoned offenders in need of short-term intervention. Treatment objectives include therapy of the mental disorder, consequent reduction of individual risk, and adequate social rehabilitation. The Cantonal Ethics Committee of Zurich evaluated this study and granted approval.

### Subjects

The subjects of this study were drawn from a sample of mentally disordered offenders who had been referred for treatment to a forensic psychiatric inpatient hospital and according to the DSM-5 [46] had been diagnosed with a schizophrenia spectrum disorder by their psychiatrist at final discharge. With this study being part of a larger research project exploring the relationship between schizophrenia and criminal offending, a subsample of patients from the original dataset (*N* = 370) was examined meeting the following criteria: (1) patients who had been referred to the forensic facility according to § 59 of the Swiss penal code (see Background for a description of the Swiss legal system) since 1990, who (2) had been discharged after successful treatment completion. Patients who were admitted for short treatment of acute syndromes (crisis intervention – length of stay under 3 months; 164 subjects), who died (1 subject) or fled from the facility (2 subjects), who were discharged because of treatment failure or transferred to another forensic facility in order to complete therapy elsewhere (27 subjects) and patients in treatment at the time of data collection (33 subjects) were excluded from the study. This left a total of 143 forensic patients meeting the inclusion criteria of this study. These strict criteria ensured presence of the same legal requirements for being released in all examined cases, and that the “true” length of inpatient treatment was considered, as recently proposed in a review of extant research [6].

The final sample studied was predominantly male (88.1%, *n* = 126) with a mean age of 34.69 years (SD 10.9). The majority of the sample was single (65.5%, *n* = 93), unemployed at the time of the offense (71.6%, *n* = 101) and born in Switzerland (54.5%, *n* = 78). 88.8% (*n* = 127) of the participants met criteria for schizophrenia, 7.7% (*n* = 11) met criteria for other schizophrenia spectrum disorders, and 3.5% (*n* = 5) met criteria for schizoaffective disorder. Length of stay ranged from the shortest hospitalization of 30 weeks to the longest of 902 weeks. The 25th percentile was 130 weeks, the median (50th percentile) 220 weeks and the 75th percentile 278 weeks.

### Data collection

A retrospective content analysis of case files for all variables was conducted using a structured protocol based on the extended [47, 48] set of criteria by Seifert [49]. On a practical level, multidisciplinary patient records compiled during patients’ hospitalization (e.g. forensic psychiatric expert reports, indictments, court judgements, nursing reports, annual reports, risk assessment reports, discharge reports, medication, etc.) were systematically reviewed and coded by a trained independent physician. To estimate inter-rater reliability, a second trained independent rater coded a random subsample of 10% of the cases. Cohen’s Kappa value [50] was 0.78, which can be considered to be substantial [51].

### Machine learning

Since the present study is explorative in nature, supervised machine learning seemed most suitable for our objectives. With supervised ML, a result (often dichotomous; e.g. ill/ not ill, short duration of stay/ long duration of stay) is defined a priori. A number of variables is used to try to distinguish between the two defined possible outcomes. ML will try to predict on the basis of these variables (e.g. socio-demographic data, symptoms) whether a possible future case (e.g. patient) can be assigned to one of the possible outcomes (e.g. ill/ not ill). The learning algorithm can also compare its result with the correct, intended result and find errors to modify the model accordingly*.* The goal of a supervised learning model is to predict the correct label for new input data using different mathematical algorithms (e.g. logistic regression, support vector machines (SVM), decision trees or k-nearest neighbor (KNN)) depending on the data structure.

The advantages compared to conventional (hypothesis testing) statistical methods are manifold: Possible hidden interrelationships in data sets can be uncovered exploratively, a large number of variables and their possible links can be examined at once, different (even non-linear) algorithms can be tested, and finally, the performance of the algorithms can be evaluated quantitatively by transcending simple *p*-value thresholds. These data-driven methods of ML have one major risk: overfitting. This means that the mathematical algorithms depend heavily on the data structure and are sensitive to “noise” within the data, which leads to overestimation in the prediction. The fewer observations and the more predictors, the higher the risk of overfitting. There are several techniques to avoid or minimize overfitting, such as cross-validation, regularization or a reduction of predictors. Nevertheless, the generalizability of ML results from one data set should be treated with caution and needs further confirmation by new data and perhaps more conservative statistical approaches.

### Statistical analysis

**Fig. 1:**
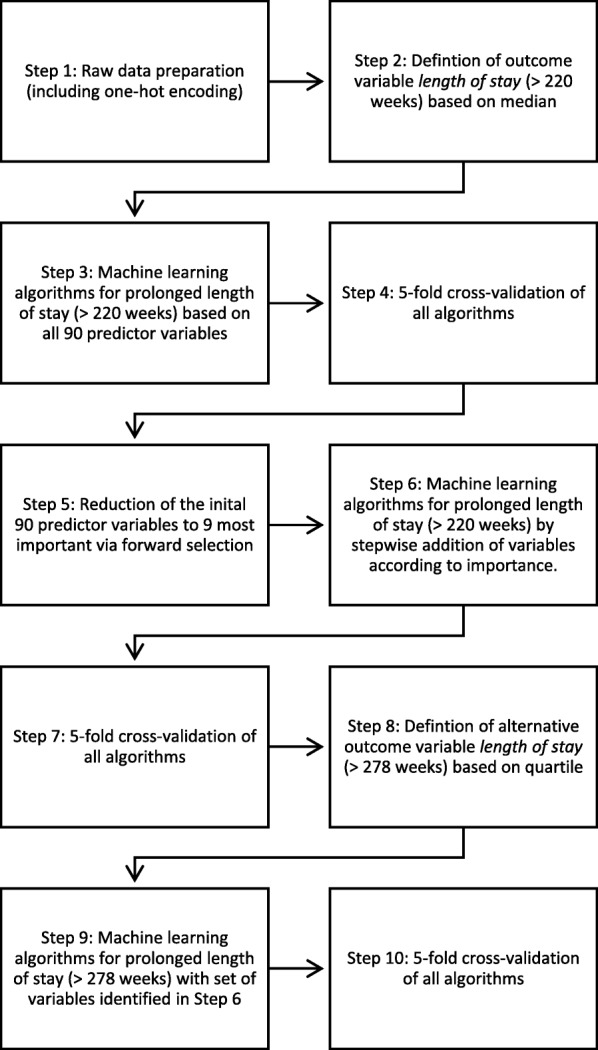
Data processing and statistical analysis

Figure 1 provides an overview of the statistical steps of our study, which are described in detail below. Algorithm selection and performance testing were conducted using MATLAB (MATLAB and Statistics Toolbox Release 2012b, The MathWorks, Inc., Natick, Massachusetts, United States.). Forward selection was performed using R Studio version 1.1.383.

#### Data preparation

All raw data was first processed for machine learning (multiple categorical variables converted to binary code) using one-hot encoding (see Fig. 1, step 1) [23, 24]. Continous variables were not manipulated.

#### Defining the outcome variable

There is considerable variance between extant studies in defining prolonged inpatient treatment [6]. Some authors defined prolonged inpatient treatment as forensic hospitalizations lasting longer than 2 years [15, 17, 30], while others used a threshold of 4 years [42], or defined the parameter as a continuous variable [1, 3, 25, 34, 35, 37].

Due to above inconsistencies defining the outcome (dependent) variable *length of stay* was difficult. To keep the complex task of ML more basic, a dichotomous subdivision seemed practical. As self-defined lengths are problematic and object to bias, we found the approach of Fong et al. [28] using the median as the outcome variable suitable. The total number of weeks between an offender patient’s admission and his or her discharge from the forensic psychiatric hospital was determined, the median calculated and prolonged hospitalization defined as lasting longer than this median number of weeks (prolonged stay, Definition 1: > 220 weeks; see Fig. 1, Step 2). ML was then performed with this first outcome variable.

According to this rationale, the results for a longer than median stay should be even more pronounced when comparing only cases with very short and very long lengths of stays. To confirm and evaluate this hypothesis, we have defined another alternative outcome variable based on the top quartile of the length of stay, which represents the prolonged stay (Definition 2: > 278 weeks; see Fig. 1, Step 8). We then repeated the last machine learning procedure with this second, alternative outcome variable.

#### Defining the predictor variables

To generate the initial set of (independent) predictor variables to be examined (see Introduction, Table 1 and Additional file 1 for a detailed description of the variables), we conducted computerized searches in various academic databases (i.e. Medline (PubMed), psychINFO, Embase, Social Sciences Citation Index (SSCI) and Google Scholar), using the following keywords in various combinations: “*length of stay*”, “*length of hospitalization*”, “*length of detention*”, “*length of admission*”, “*offenders*”, “*mentally ill*”, “*forensic*”, “psychiatr*”, “*hospital*”, and “*mental health services*”. For the purpose of retrieving additional literature, citation indices were used for a forward search. A backward search was carried out by viewing the provided references of selected materials. With regards to inclusion/exclusion criteria, only academic contributions (i.e. peer-reviewed articles, books, and conference proceedings) in English and German were considered, which examined the length of stay of forensic psychiatric patients as dependent variable. No restrictions were imposed to the time frame, country, or region of the studies. All variables explored in these identified studies were considered as possible predictor (independent) variables. A small amount of these variables could not be examined due to high rates of missing values in our data (e.g. HCR, PCL) or due to the uniqueness of the specific item (e.g. DUNDRUM scores).

#### Machine learning and model evaluation

For statistical analyses, supervised ML was first performed with all 90 possible predictor variables to find the algorithm (the model) with the best predictive accuracy for Definition 1 of the outcome variable *length of stay* (prolonged length of stay > 220 weeks; see Fig. 1, Step 3). With 143 observations and 90 predictors ML is susceptible to overfitting. To counteract this problem and ensure good predictive performance of an algorithm, the most common approach to estimating prediction error is cross-validation. Cross-validation refers to techniques that involve training and testing an algorithm on different subsamples of the whole dataset [52]. To this end, the entire data set of the present study was divided into five equally sized subsets (5-fold cross-validation), with four subsets being used for training all algorithms subsequently examined and the remaining subset for evaluating the accuracy of the algorithms (see Fig. 1, Step 4). Cross-validation was also used for all following ML steps (see Fig. 1, Steps 7 and 10). Algorithms deemed accurate after cross-validation were chosen for further evaluation of their performance: Goodness of fit was assessed using the receiver operating characteristic (ROC) curve method [53]. Area under the curve (AUC) served as the criterion to determine the level of discrimination. Additionally, specificity and sensitivity, positive predictive value (PPV) and negative predictive value (NPV) were calculated.

The next task was to identify the most important of the 90 predictor variables, to quantify their influence on the model and to reduce the algorithm’s susceptibility to overfitting. Forward selection [54], a technique based on subset selection (a statistical regression method utilized to find a small subset of available predictor variables that are most relevant for predicting the outcome variable), was used to reduce the number of predictor variables to a subset of their most predictive 10% (see Fig. 1, Step 5). The resulting nine variables were then ranked according to their importance as identified by the forward selection method. In addition, their *p*-values were derived via Fisher’s exact tests or Mann-Whitney U-tests.

The same machine learning procedure, cross-validation and performance assessment as described above was then repeated with each of the 9 variables identified by the forward selection method and their combinations (Fig. 1, Steps 6 and 7). Thus, a total of nine to the power of 9 combinations of the 9 most predictive variables were tested in a stepwise manner. The goal of this was to find an algorithm based on only as many prediction variables as necessary to achieve an AUC similar to that in the algorithm based on all 90 predictor variables. Finally, all steps taken for the statistical analysis based on the 9 variables identified so far by forward selection were repeated for the second definition of the outcome variable *length of stay* (Definition 2: extended hospital stays > 278 weeks; see Fig. 1, steps 9 and 10).

## Results

The performance and composition of the predictor variables of the algorithms that best predict the first definition of the outcome variable *length of stay* (hospitalization of more than 220 weeks) are presented in Table 2 and the variable importance identified by forward selection is shown in Table 3. The first algorithm, which considered all possible predictor variables, identified boosted trees as the most accurate statistical analysis procedure yielding an AUC of 0.67. Algorithms based solely on the predictor variable “victim injured severely/ fatally” (statistical procedure: boosted trees) or “index crime: (attempted) homicide” (statistical procedure: KNN) both resulted in an AUC of 0.60, which corresponds to 89.55% of the AUC of the algorithm based on all 90 predictor variables. The combination of these two variables in an algorithm yielded an AUC of 0.65 (no multicollinearity; statistical procedure: SVM) which corresponds to 97.01% of the AUC of the algorithm based on all 90 predictor variables. All other nine to the power of nine algorithms explored based on the nine most predictive predictor variables or combinations thereof (see Table 3) led to negligible AUCs ranging between 0.48 and 0.52. Likewise, only the *p*-values of the variables “seriously/ fatally injured victim” and “index crime: (attempted) homicide” were significant, confirming these variables as the most important (see Table 3). In summary, the model using only the two variables associated with index crime seemed the most suitable to achieve an acceptable AUC and minimize overfitting. This model had a sensitivity of 63%, reflecting its ability to correctly classify the actual “long stay” cases, and a slightly higher specificity of 68%, indicating its ability to correctly identify those with “short stay”. The probability that the persons identified by the model as having a “long stay” are in fact staying longer than the median of all stays (PPV) was 75%. The probability that the persons the algorithm identified to belong to the “short-stay”-group were actually staying shorter than the median (NPV) was 55%.

**Table 2:** Model selection for outcome variable length-of-stay by median

Variable	Best algorithm/ statistical procedure	Accuarcy (%)	AUC	Sensivity (%)	Specificity (%)	PPV (%)	NPV (%)
All Variables (90)	Boosted trees	65	0.67	63	69	76	54
Victim injured severly/fatally	Boosted trees	65	0.60	63	68	75	55
Index crime: (attempted) homicide	KNN	61.5	0.60	61	62	61	62
Victim injury severly/fatally and index crime (attempted) homicide	SVM	65	0.65	63	68	75	55

**Table 3:** Distribution of predictor variables by importance after forward selection

variable	short length-of-stay n/N (%)	long length-of-stay n/N (%)	***p***-value*
1. Victim injured severly/fataly	18/60 (30)	39/68 (68.4)	0.002
2. Index crime: (attempted) homicide	19/72 (26.4)	33/71 (46.5)	0.015
3. Index crime: sexual abuse of children	0/72 (0)	3/71 (4.2)	0.120
4. Selfharming during current hospitalization	10/70 (14.3)	6/71 (37.5)	0.301
5. Index crime: threat, coercion	27/72 (37.5)	22/71 (31)	0.482
6. Index crime: property crime with violence	3/72 (4.2)	4/71 (4.9)	0.719
7. PANSS at admission (mean, SD)	23 (11.89)	25.61 (12.90)	0.277
8. Experience of poverty in childhood/adolescence	25/63 (39.7)	22/62 (35.5)	0.713
9. Hallucinations described in past psychiatric history	40/72 (55.6)	46/71 (64.8)	0.307

Note. *SD* Standard deviation

* *p*-value derived from Fisher’s exact test; *p*-value variables “PANSS at admission” derived from Mann-Whitney-U-test

The algorithms that best predicted the second definition of outcome variable *length of stay* (hospital stays of more than 278 weeks) produced similar results, which are presented in Table 4. Consequently, the algorithm based solely on “ victim injured severely/ fatally” resulted in an AUC of 0.64 and the algorithm based on “index crime: (attempted) homicide” yielded an AUC of 0.59. A combination of both variables led to an increased AUC of 0.71, a sensitivity of 78% and a specificity of 79%. PPV and NPV showed no alteration.

**Table 4:** Model selection for outcome variable laytime by quartile

Variable	Best algorithm/ statistical procedure	Accuarcy (%)	AUC	Sensivity (%)	Specificity (%)	PPV (%)	NPV (%)
Victim injured severly/fatally	Bagged trees	73.6	0.64	70	79	83	64
Index crime: (attempted) homicide	KNN	65.3	0.59	62	70	78	53
Victim injury severly/fatally and index crime (attempted) homicide	SVM	73.6	0.71	78	79	75	55

## Discussion

The aim of this study was to investigate the role of a large number of previously researched factors that may affect the length of forensic inpatient treatment of offender patients with schizophrenia spectrum disorder. Using machine learning algorithms, it was possible to detect important influencing factors. The final model identified serious index offences such as homicides and the severity of injuries inflicted on the victim of the offence as the two parameters most closely related to the length of forensic hospitalization. With an AUC of 0.65, a sensitivity of 63% and a specificity of 68%, a correct long or short stay could be determined in two thirds of the cases. When considering extreme values using the 75th percentile, the model performed even better with an AUC of 0.71 and about 80% of patients could be correctly identified as staying longer or shorter. Results are consistent with prior research identifying the severity of the index offence as a major factor [25, 35, 40, 41] or at least a factor of partial relevance [1, 6, 14–17, 28–34, 36–39, 41, 55] in explaining prolonged forensic inpatient treatment. This study confirms these findings specifically for offender patients with a schizophrenia spectrum disorder. In contradiction to previous studies [1, 3, 6, 7, 14–17, 29, 31–34, 36, 38, 39, 41–44, 55], however, ML did not confirm sociodemographic factors, other aspects of the criminological or psychiatric patient history, further treatment related, or psychopathological factors to affect the length of forensic inpatient treatment in our sample of patients. In other words, the length of forensic inpatient treatment was determined by factors seemingly invariable by therapeutic efforts. One explanation may be that the crimes of offender patients with prolonged forensic hospitalizations in this study blinded institutions involved in patients’ assessment and treatment (investigative authorities, courts of law, clinicians, enforcement agencies) to such an extent, that positive treatment effects allowing an earlier release were (partially) ignored. Barriers to being released may have been higher for patients committing more severe crimes than to those responsible for less profound criminal behavior. Clinicians and courts of law may feel responsible for the prevention of similarly severe crimes under all circumstances in the future. Also, political considerations for public safety and the individual views of clinical and public decision-makers on risk assessment may prevent treatment initiatives, possibly influenced by unobjective media coverage about schizophrenic offenders. This zero-risk mentality would overlook the question of whether the risk of recidivism can and must be countered by mechanisms other than long-term hospitalization. Positive developments in offender patients, which would warrant a release from forensic inpatient treatment in cases of less severe crimes, may be mistrusted in cases with severe index offences. Despite that forensic psychiatry should not base treatment on the severity of index offences alone, but rather on risk assessments, this seems to be difficult in criminal cases where emotions can be expected to be high due to the cruelty of a crime. However, this study did not explore if offender patients with prolonged inpatient treatment were also considered to be of high risk for reoffending. Assessing the future risk of recidivism in forensic patients is a complex task that is difficult to operationalize in parameters (such as criminal risk assessment tools or verbalized treatment effect scores) that are valid for further testing of the above hypothesis.

Another explanatory approach may be that if aftercare conditions do not seem optimal, clinicians are somewhat hesitant to recommend release. Only a few Swiss cantons have specialized and sufficiently developed aftercare services. This entails the risk that the patients’ progress achieved in inpatient treatment will dissipate under everyday conditions.

Future research should therefore not be limited to a collection of patient factors, but rather examine individual dynamic treatment processes and also include qualitative clinical data. More research is also needed on the various aspects of aftercare for released offenders, as effective aftercare may reduce the risks associated with discharge and may contribute to increasing the number of patients considered suitable for release.

The results presented here provide some thought-provoking insights, since psychiatric patients are apparently exposed to factors that are too complex to be easily measured and influenced. Novel statistical approaches such as ML can help bring clarity into these complex variable relationships and uncover previously hidden relationships, confounders and intermediates.

### Limitations

The present analysis was based on retrospectively collected data with its known analytical problems. Although the files used in this study were extensive and the information was of high quality, distortions in the medical files could not be completely excluded and, in addition, complex variables had to be reduced to a simple dichotomous response resulting in loss of information.

ML achieves particularly good results with large data sets. The 143 patients analysed remain a small quantity in this context and so, despite cross-validation, overfitting remains a limitation to the interpretability of this study.

## Conclusion

The present study identified factors associated with prolonged inpatient treatment (> 220 weeks or > 278 weeks) in offender patients diagnosed with a schizophrenia spectrum disorder, who were admitted to a Swiss forensic hospital in order to reduce their risk for criminal recidivism. Factors identified as relevant in extant research were explored using a novel statistical methodology more apt to reveal non-linear or confounding interdependencies between variables thus aiming to address inconsistencies in prior research results. Criteria related to the index offense had a significant impact on prolonged duration of inpatient forensic psychiatric treatment.

## Supplementary information

**Additional file 1.** Description of variables explored in current study.

## Data Availability

The datasets used and/or analysed during the current study are available from the corresponding author on reasonable request.

## Abbreviations

PANSS: Positive and Negative Syndrome Scale
DSM-5: Diagnostic and Statistical Manual of Mental Disorders, Fifth Edition
SD: Standard deviation
HCR: Historical, Clinical and Risk Management
PCL: Psychopathy Checklist
DUNDRUM: Dangerousness, Understanding, Recovery and Urgency Manual
ML: Machine Learning
ROC: Receiver operating characteristic
AUC: Area Under the Curve
KNN: k-Nearest-Neighbour SVM Support Vector Machine
PPV: Positive Predictive Value
NPV: Negative Predictive Value
